# Performance determinants in Ski mountaineering sprint: data from the pre-2026 Olympic test event

**DOI:** 10.3389/fspor.2026.1833475

**Published:** 2026-06-24

**Authors:** Justin Mottet, Forrest Schorderet, Antoine Raberin, Grégoire P. Millet, Nicolas Bourdillon

**Affiliations:** 1 Institute of Sport Sciences (ISSUL), University of Lausanne, Lausanne, Switzerland; 2 Swiss Ski Mountaineering Federation, Swiss Alpine Club, Bern, Switzerland; 3 Teaching and Research Unit in Physical Education and Sport (UER EPS), University of Teacher Education (HEP-VD), Lausanne, Switzerland

**Keywords:** aerobic power, lactate kinetics, Olympic games, pacing strategy, Ski mountaineering

## Abstract

**Purpose:**

Ski mountaineering (SkiMo) sprint is a new Olympic discipline. This study aimed to investigate the performance determinants at the Milan-Cortina Olympic Sprint Test Event through the analysis of overall sprint time in quarterfinals (SPT), section-specific contributions, and associations with laboratory-derived physiological parameters.

**Methods:**

SPT and seven section splits were analyzed across quarterfinal rounds in qualified athletes (*n* = 36). The course included uphill (U), transition (T), and downhill sections. A subgroup of Swiss elite male sprinters (*n* = 9, Tier 4–5) underwent laboratory testing of aerobic power and lactate kinetics.

**Results:**

Time on uphill ski and foot section (UT) in quarterfinals accounted for the largest proportion of SPT variability (81.5%) and was strongly correlated with SPT (*r* = 0.89, *P* < 0.001), particularly during U1, the first U section of the race (*r* = 0.91, *P* < 0.001).

Time in T1 showed a moderate association with SPT (*r* = 0.62, *P* = 0.021), whereas no significant associations were observed for the remaining U, T, or downhill sections time.

Among the top 18 of the 36 qualified athletes, T time contributed more to SPT variability than in athletes ranked 19th to 36th (respectively 46.8% and 18.4%).

For the subgroup tested in the laboratory, UT was best predicted by maximal vertical velocity (*r* = −0.76, *P* = 0.018) and by maximal oxygen uptake relative to body mass ($\dot{V}O_{2\max}$) (*r* = −0.75, *P* = 0.021), and a multiple stepwise regression identified $\dot{V}O_{2\max}$ and lactate removal capacity as the best UT predictors (*R*^2^ = 0.82, *P* = 0.006).

**Discussion:**

These findings suggest that sprint performance in SkiMo is associated with both high aerobic capacity, and strong performance in the early sections of the race (U1 and T1). Interestingly, lactate removal capacity was also associated with sprint performance, suggesting a potential role of peripheral muscle characteristics associated with lactate kinetics on fatigue resistance during a SkiMo sprint. Finally, the relatively greater contribution of T to sprint performance among world-class athletes highlights that SkiMo sprint is not only about physiological capacity but also about fast and error-free T under maximal effort.

## 1 Introduction

**Figure 1:**
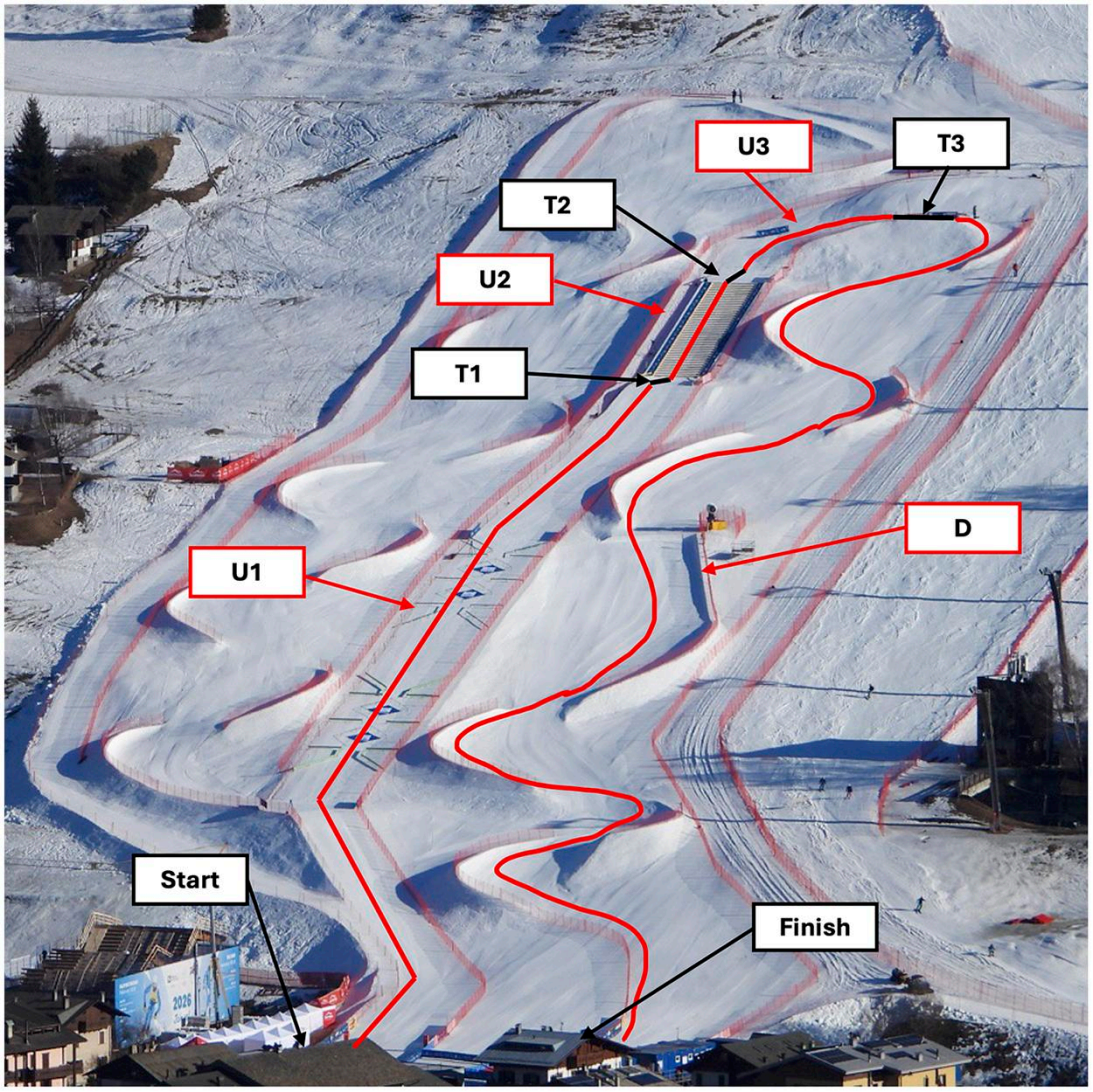
Sprint course and selected sections from the 2025 milan-cortina Olympic test event, identical to the course used at the 2026 winter Olympic games.

Ski Mountaineering (SkiMo) is a new winter Olympic sport that made its debut at the Milano Cortina 2026 Olympic Games. SkiMo is an endurance sport performed on snow-covered terrain. Athletes climb uphill using skis with climbing skins and poles, with a technique similar to the diagonal stride technique in cross-country skiing but on steeper slopes. For downhill sections, the heel is fixed to the ski, allowing an alpine-ski-like descent. Several race formats vary in distance, duration, and elevation gain, ranging from traditional disciplines such as team race, individual race, and vertical race to the two disciplines selected for the 2026 Olympic Games: mixed relay and sprint (1). The SkiMo sprint consists of multiple high-intensity efforts lasting approximately 2.5–3 min on a course with about 70 m of ascent. The course includes two uphill skiing sections (U1 and U3) separated by a very steep foot section (U2) and followed by a downhill skiing section to the finish (D) (Figure 1). Transitions (T) occur between sections to switch locomotion modes: the first (T1) involves placing the skis on the backpack to start the foot section, the second (T2) consists of putting the skis back on for the second uphill skiing section, and the third (T3) involves removing the skins and switching from an uphill to a downhill skiing setup (Figure 1). A SkiMo sprint competition begins with a time-trial qualification, followed by three knockout heats in which athletes are progressively eliminated. Qualified athletes complete one lap of the course in each heat, with 20–25 min of recovery between rounds (2).

From a field performance perspective, several descriptive data have been reported in SkiMo sprint, notably the minimal time differences relative to the race winner required to qualify for the next round or to secure a podium. In the 2022–2023 season, the thresholds to qualify for the quarterfinals (QF), semi-finals (SF), finals (F), and to reach the podium were approximately 18%, 9.5%, 8%, and 5% slower than the race winner's time, respectively (3). The best athletes also tended to improve their performance from the heats to the finals (3). Fornasiero et al. (4) also found that during a sprint, U time accounts for ∼64% of sprint time, T – 17%, and D ∼ 8%. They also reported that, in a stepwise regression analysis, uphill (U) time alone including U1, U2, U3 explained 80%–90% of the variance in sprint time and after adding T to the model, the explained variance increased to 95%–98%. Wagner et al. (5) also reported a moderate correlation between sprint time and T time.

In this context, where U excluding T and D lasts – ∼1.5–2 min, the best laboratory predictors in men were maximal sustainable speed over 2 min and maximal vertical speed (vV_max_) at the end of a graded exercise test to exhaustion (GXT) (6). Absolute oxygen consumption ($\dot{V}O_{2\max}$) was moderately to strongly correlated with U only in men. Moreover, Wagner et al. (5) reported associations with whole-body and upper-body relative and absolute $\dot{V}O_{2\max}$, vV_max_, fat-free mass, and maximal strength in a junior sample including both males and females, accounting for a potential sex effect.

Analyses of similar race formats, such as cross-country skiing sprints, indicate that aerobic parameters are strong predictors of performance (7, 8). Moreover, the importance of aerobic capacity appears to increase across consecutive heats (9), consistent with the substantial aerobic contribution reported for this type of effort (10). Nevertheless, anaerobic metabolism also plays a significant role, as short uphill sections can be performed at intensities ∼60% above aerobic capacity (8), and approximately 24% of the total energy contribution during a full heat has been attributed to anaerobic sources (10). In addition, gross efficiency and maximal speed have been significantly associated with sprint performance in cross-country skiing (8). Finally, World Cup (WC) skiers have been shown to exhibit greater active blood lactate recovery and longer time to exhaustion at maximal speed compared with national-level skiers (7).

In SkiMo, similar to what has been studied in cross-country skiing, performance characteristics and physiological determinants should be investigated more extensively in World best athletes. The present study was designed to investigate (a) race section contributions to sprint time during QF (SPT) across different performance-level groups, and (b) the greatest laboratory and physiological predictors of uphill time (UT) in QF calculated by adding the times of the two ski sections with the time of the foot section (U1 + U2 + U3) without the T times.

## 2 Methods

### 2.1 Overall design of the study

During the summer preparatory period, athletes from the Swiss national team completed a comprehensive laboratory testing battery. This assessment included:

1. maximal performance and associated physiological variables obtained during a graded exercise test (GXT) performed at a 25% incline;
2. lactate kinetics assessed throughout the 20 min recovery period following the GXT; and
3. supramaximal performance and corresponding physiological variables measured during a time-to-exhaustion test performed at 120% of the maximal speed achieved during the GXT (TTE).

Eight months after the laboratory testing, performance during the Milan-Cortina Olympic sprint Test Event 2025 (OSTE), part of the International Ski Mountaineering Federation (ISMF) WC, was analyzed. SPT and time spent in seven predefined race sections were quantified. In addition, the ISMF sprint points for the full 2025 season were collected for each athlete. ISMF sprint points were calculated as the sum of points earned by each athlete in all sprint competitions throughout the season, obtained from the official ISMF results website.

Two main analyses were conducted:

1. QF performance analysis aimed at evaluating the relative contribution of each race section to overall SPT on the OSTE course; and
2. analysis of the relationships between UT in QF and laboratory-measured physiological parameters.

### 2.2 Subjects

**Figure 2:**
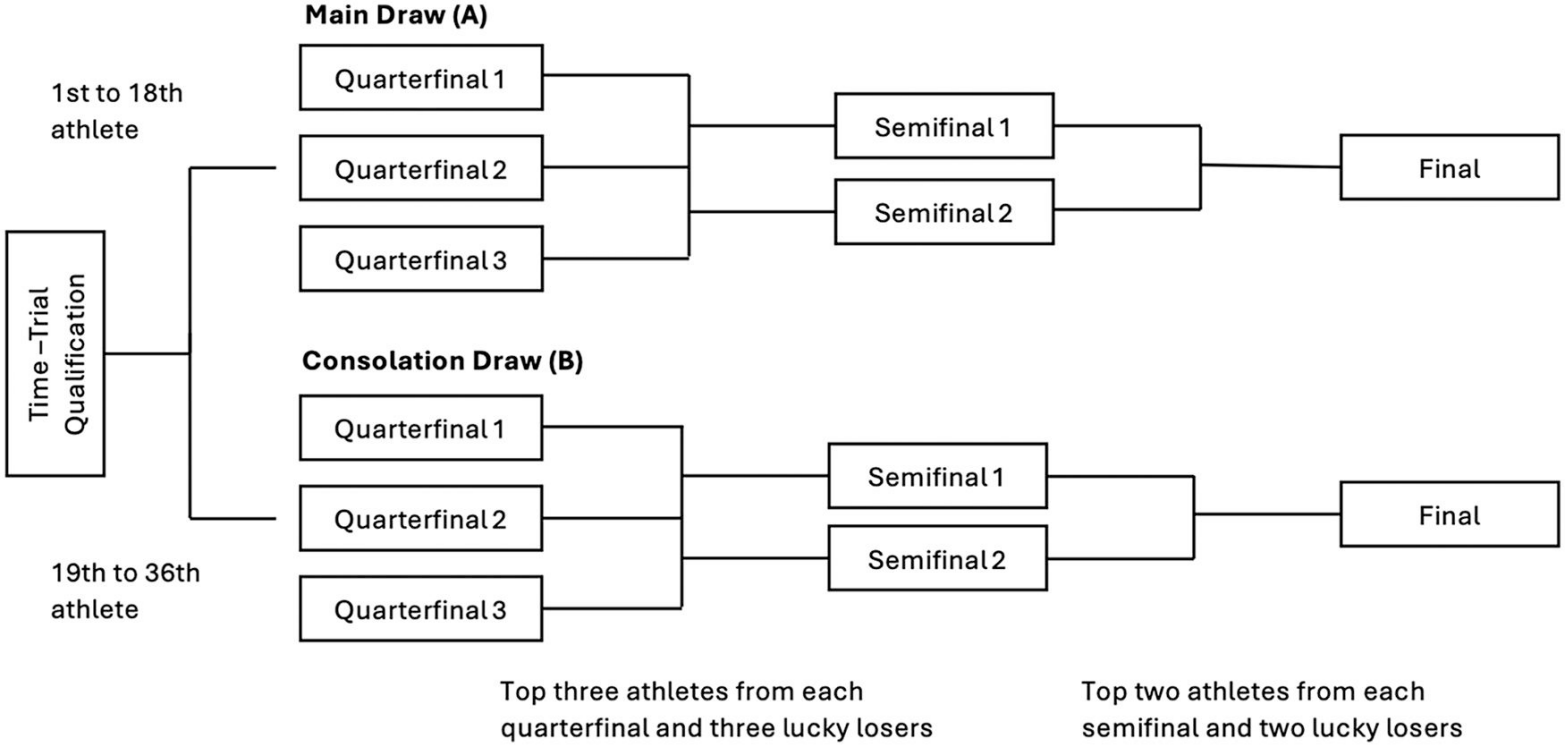
Milan-Cortina test event 2025 competition format.

1. For QF performance analysis, the first 36 men athletes according to the official time-trial qualification of OSTE were initially included (Figure 2). The top 18 athletes were assigned to the QF A and the next 18 to the QF B. To minimize potential motivational bias, only athletes who qualified for SF in each QF were retained for analysis. Consequently, the final sample consisted of 24 athletes (12 from the QF A and 12 from the QF B).
2. For the physiological analysis, 10 male sprint SkiMo athletes classified as *elite* (*n* = 5) or *world-class* (*n* = 5) according to McKay et al. (11) and members of the Swiss national team were initially recruited. The world-class category includes Olympic and World Championship, and Wold cup medalists, whereas the elite category comprises athletes who also compete at the international level but did not reach a top-3 ranking. One athlete was excluded from the statistical analysis due to atypical performance attributed to illness on the race day. The remaining athletes were aged 20–32 years and had all previously competed in multiple SkiMo sprint and mixed-relay WC events. Their competitive achievements included one Olympic medalist, two World Champions, three World Championship medalists, four WC winners, six WC medalists, and three European Championship medalists. Their characteristics are presented in Tables 1, 2. All athletes were informed about the study procedures and provided written informed consent prior to participation. The study was approved by the institutional ethics committee (CER-VD 2023–01638) and conducted in accordance with the Declaration of Helsinki.

**Table 1:** Anthropometric and sprint performance characteristics of the 9 ski mountaineering skiers (tier 4–5) involved in physiological analysis.

Variables	Quarterfinal A (*n* = 4)	Quarterfinal B (*n* = 5)	Overall (*n* = 9)
Age (years)	21.5 ± 3.0 (20–26)	26.4 ± 3.8 (22–32)	24.2 ± 4.1 (20–32)
Body height (cm)	180.3 ± 6.8 (174–190)	183.4 ± 6.9 (176–193)	182.0 ± 6.7 (174–193)
Body mass (kg)	68.7 ± 8.2 (61.4–80.4)	72.2 ± 8.4 (64.4–85.2)	70.6 ± 8.0 (61.4–85.2)
Body mass index (kg.m^−2^)	21.1 ± 0.9 (20.3 −22.3)	21.4 ± 1.0 (20.1–22.8)	21.2 ± 0.9 (20.1 −22.9)
ISMF sprint points	415.8 ± 93.3 (302–527)	163.6 ± 66.5 (105–251)	275.7 ± 152.1 (105–527)

**Table 2:** Laboratory-based physiological and performance parameters of the 9 ski mountaineering skiers (tier 4–5).

Variables	Quarterfinal A (*n* = 4)	Quarterfinal B (*n* = 5)	Overall (*n* = 9)
Graded exercise test			
vV_max_ (m.h^−1^)	2,145 ± 128 (1,975–2,275)	1,990 ± 63 (1,900–2,050)	2,058 ± 121 (1,900–2,275)
$\dot{V}O_{2\max}$ (L.min^−1^)	4.91 ± 0.43 (4.55–5.45)	4.88 ± 0.69 (4.27–6.04)	4.89 ± 0.55 (4.27–6.04)
$\dot{V}O_{2\max}$ (mL.min^−1^.kg^−1^)	73.4 ± 3.1 (69.9–77.2)	68.1 ± 2.0 (65.9–70.9)	70.5 ± 3.7 (65.9–77.2)
$\dot{V}E_{\max}$ (L.min^−1^)	180.2 ± 8.7 (173.2–191.3)	192.3 ± 17.2 (176.8–220.7)	186.9 ± 14.7 (173.2–220.7)
HR_max_ (bpm)	189.5 ± 2.6 (187–193)	191.8 ± 2.8 (189–195)	190.8 ± 2.8 (187–195)
RER_max_	1.02 ± 0.04 (0.98–1.08)	1.07 ± 0.06 (0.98–1.14)	1.05 ± 0.06 (0.98–1.14)
BLa_max_ (mmol.L^−1^)	13.4 ± 2.2 (11.0–16.3)	12.4 ± 2.0 (9.7–14.9)	12.8 ± 2.0 (9.68–16.28)
ΔBLa_rec_ (mmol.L^−1^)	5.6 ± 1.3 (4.2 −7)	5.5 ± 1.1 (4.1–7)	5.5 ± 1.1 (4.1–7)
ΔBLa_rec_ (%)	43.7 ± 15.5 (25.7–57.1)	44.8 ± 7.5 (35.3–51.7)	44.3 ± 10.9 (25.7–57.1)
Time to exhaustion test			
TTE test speed (m.h^−1^)	2,550 ± 194 (2,280–2,730)	2,388 ± 75 (2,280–2,460)	2,460 ± 156 (2,280–2,730)
TTE test time (s)	161.8 ± 26.6 (130–187)	153.2 ± 19.4 (140–187)	157.0 ± 21.7 (130–187)
BLa_preTTE_ (mmol.L^−1^)	7.8 ± 3.4 (4.7–12.9)	6.9 ± 1.6 (4.7–9.2)	7.3 ± 2.4 (4.7–12.1)
BLa_maxTTE_ (mmol.L^−1^)	16.8 ± 3.0 (13.54–19.95)	14.3 ± 1.6 (13.2–17)	15.4 ± 2.5 (13.2–20)
ΔBLa_TTE_ (mmol.L^−1^)	9.0 ± 1.2 (7.9–10.3)	7.4 ± 1.2 (5.7–8.5)	8.1 ± 1.4 (5.7–10.3)

vV, vertical velocity; V̇O_2_, oxygen uptake; V̇E, ventilation; RER, respiratory exchange ratio; BLa, blood lactate concentration; ΔBLa_rec_, 20 min passive lactate recovery; Max, maximal effort; TTE, time to exhaustion; BLa_preTTE_, blood lactate concentration before TTE test; BLa_maxTTE_, maximum blood lactate concentration after TTE test, ΔBLa_TTE_, blood lactate increase during the TTE test.

### 2.3 Analysis of the Olympic sprint test event

#### 2.3.1 Competition course description

During the official course inspection, conducted approximately 20 min before the time-trial qualification, the sprint course and its individual sections were measured using a real-time kinematic GPS system (Innove, Switzerland). Owing to organizational constraints, we were not permitted to follow the exact D racing line during the inspection in order to prevent damage to the course; therefore, a precise characterization of the D section could not be performed.

The OSTE was held on a course identical to that used for the 2026 Olympics, located at an altitude of 1217 m above sea level, comprising a total uphill distance of 314.7 m with an elevation gain of 73.6 m, followed by a downhill section with a corresponding elevation loss.

The course was divided into seven distinct sections with different properties (Figure 1; Table 3). Three sections corresponded to T phases (T1, T2, T3), during which athletes manipulated their bindings, skis, and skins. Two sections consisted of U skiing (U1, U3), one section was a U portage on foot with skis carried in a backpack (U2), and the final section was a short ski-cross downhill segment (D).

**Table 3:** Time spent in the seven track sections during the quarterfinal and its correlation with SPT in 24 elite male ski mountaineering athletes who subsequently qualify for the semi-final.

Terrain	Track section	Technique	Section length (m)	Section decline (%)	Time in section (s)	Speed (m.s^−1^)	Vertical velocity (m.h^−1^)	Correlation between section time and SPT
Uphill	U1	SU	247.2	22.8	76.51 ± 4.78	3.24 ± 0.2	2,667 ± 166	0.91****
	U2	FA	29.3	31.9	12.96 ± 0.85	2.27 ± 0.15	2,614 ± 175	0.48
	U3	SU	20.7	31.6	9.14 ± 0.78	2.28 ± 0.19	2,593 ± 214	0.44
	Total		297.2	24.4	98.62 ± 6.29	3.02 ± 0.17	2,650 ± 146	0.89****
Transition	T1	M1			9.21 ± 0.72			0.62*
	T2	M2			8.67 ± 0.79			0.13
	T3	M3			12.87 ± 1.23			0.33
	Total				30.75 ± 1.97			0.48
Downhill	D	DW			33.39 ± 0.98		−7,946 ± 231	0.22

U, Section involving uphill displacement; T, Section involving technical manipulation; SU, Skiing uphill movement; FA, Foot ascent with skis on the backpack; DW, Descent while skiing; M1, putting the skis on the backpack; M2, putting the skis on; M3, taking skins off; SPT, Total sprint time in the quarterfinals.

* *P* < 0.05.

** *P* < 0.01.

*** *P* < 0.001.

**** *P* < 0.0001.

#### 2.3.2 Competition format

The OSTE followed a sprint competition format, including a Q round, QF, SF, and F, with each heat contested by six athletes. However, this competition format deviated from the standard sprint WC structure, as it included a main draw with the top 18 athletes and a consolation draw for athletes ranked 19th to 36th. The allocation of athletes across the different heats is illustrated in Figure 2.

Between each heat, athletes had a recovery period of approximately 20–25 min before the next race, except after the qualifying round, when recovery lasted at least one hour.

#### 2.3.3 Time measurements

SPT and section-specific split times were provided by the official timing system (MSO, Switzerland). The system uses two electronic transponders per athlete, positioned above the shoes on both legs. These transponders were detected at the beginning and end of each course section, allowing precise split-time measurements.

For performance analyses, two different approaches were used:

1. For the QF performance analysis, SPT and split times from the QF were analyzed for athletes who subsequently qualified for the SF.
2. For the physiological analysis, only athletes who also completed laboratory testing were included, and performance was quantified using the UT. UT was defined as the sum of the U sections (U1 + U2 + U3), excluding T times. This approach was selected to minimize the influence of transition-related variability and to better reflect the athletes' underlying physical capacities.

### 2.4 Laboratory testing

A subgroup of nine Swiss athletes performed a laboratory test consisting of a GXT to exhaustion, followed by 20 min of recovery and a TTE test at 120% of the end speed reached during the GXT. All procedures related to the GXT and lactate kinetics during the recovery period have been described in detail in a companion article (12) and are only briefly summarized here for clarity. The TTE at 120% and its associated measurements, which were not presented in the companion article, are fully reported in the present study.

#### 2.4.1 Graded exercise test to exhaustion and recovery measurements

Athletes performed a walking-to-running GXT to exhaustion on a motorized treadmill set at a constant 25% slope (Cosmed T170, Rome, Italy), with speed increasing by 0.3 km/h each minute until volitional exhaustion. During the test participants used SkiMo poles. vV_max_ was defined as the last completed stage.

Gas exchange was measured breath-by-breath using a calibrated metabolic system (Quark CPET, Cosmed, Italy). $\dot{V}O_{2\max}$ was defined as the highest 20 s averaged value, and ventilatory thresholds were determined using standard visual criteria (13).

A standardized seated recovery period followed the GXT, during which heart rate and capillary blood lactate were repeatedly measured. Lactate concentration was assessed from fingertip capillary samples using an enzymatic analyzer (Biosen C-Line Clinic, EKF Diagnostics, UK). The maximal post-exercise lactate value (BLa_max_) was defined as the highest concentration observed during recovery. Lactate recovery was calculated as the difference between BLa_max_ and the value measured after 20 min of recovery (ΔBLa_rec_). This approach was selected because of the near-linear BLa decrease in our subjects (mean *R*^2^ = 0.98; lowest individual *R*^2^ = 0.90) and the short windows analysed (1–20 min post exercise) not recording the late recovery lactate clearance slowdown. No significant early recovery increase phase was observed. Seven of nine athletes reached BLa_max_ at 1 min and only two at 3 min but with a BLa_max_ value very close to their 1-min value. Although previous work has shown that lactate kinetics are well described by a bi-exponential model (14, 15), the early phase of lactate removal (1–20/30 min) is well approximated by a linear decline (16, 17).

#### 2.4.2 Time to exhaustion test

After the 20 min seated recovery, the athlete performed a TTE test at 120% of the end speed reached during the GXT, on a treadmill set at a 25% incline. Prior to this effort, a 3 min walk at 4 km/h (1,000 m/h) was performed as a standardized warm-up. No poles were used during this test. Athletes were instructed to continue running until exhaustion and were secured with a safety harness. TTE was recorded. Following the TTE, the athlete remained seated, to determine the maximal lactate concentration (BLa_postTTE_). Blood lactate concentrations were measured at 1, 3, 6, 9, 12, 16, and 20 min. The highest value recorded during this period was reported. Measurements were performed using the same procedure described above and in Schorderet et al. (12).

### 2.5 Statistical analyses

#### 2.5.1 Quarterfinal performance analysis

The Shapiro–Wilk test was used to assess the normality of all variables. Statistical significance was set at *p* < 0.05. To compare mean values between performance groups, either an independent *t*-test or the Mann–Whitney *U*-test was used, depending on the distribution of the data.

Correlations between race sections or ISMF sprint points and SPT were analyzed using Pearson's or Spearman's correlation coefficient, depending on data distribution. For non-normally distributed data, Spearman's rank correlation was used; in the presence of tied values, the *p*-value was calculated using a normal approximation, in accordance with R's default method. To account for multiple comparisons, Bonferroni correction was applied across correlations between race sections and SPT.

The contribution of split times to SPT variability was analyzed by calculating the ratio of the covariance between each section and SPT to the variance of SPT, expressed as a percentage. Analyses were performed separately for the QF A group, the QF B group, and the entire sample. In addition, the temporal contribution of each section was expressed as a percentage of SPT and is reported as mean ± standard deviation.

#### 2.5.2 Relationship between physiological parameters and uphill ski and foot section time

Correlations between the physiological parameters and UT in QF were analyzed using Pearson or Spearman's product-moment correlation coefficient test and simple linear regression was used to draw trend lines.

Stepwise multiple linear regression was used to predict UT in QF from physiological parameters. Model selection was based on a combination of Akaike Information Criterion, Bayesian Information Criterion, coefficient of determination (*R*^2^), adjusted *R*^2^, and the statistical significance of regression coefficients. Multicollinearity was assessed using variance inflation factors (VIF), and potential interaction effects were tested. Regression results are presented as both unstandardized and standardized coefficients.

To evaluate which parameters have the greatest influence on model output, a sensitivity analysis was performed on the best predictive model. All predictor variables were first standardized (*z*-scores) to allow comparison of effects across different scales. Sensitivity analysis was conducted by systematically perturbing each predictor variable by ±0.5 and ±1 standard deviation while keeping the other variables constant. For each perturbation, predictions of UT in QF were recalculated, and changes in the predicted mean and standard deviation were recorded. The relative variation in UT in QF (%) compared to the original model was then computed for each modification. Statistical analyses were performed using RStudio (version 2021.09.0; Posit, PBC, Boston, MA, USA).

## 3 Results

### 3.1 Quarterfinal performance analysis

SPT in QF was strongly correlated with ISMF sprint points (*r* = −0.77, *P* < 0.001), with a mean SPT of 162.8 ± 6.3 s (153.8–172.5) across all skiers, while the mean UT was 98.6 ± 5.5 s (91.7–108.3).

Across QF, UT was strongly correlated with SPT. When analyzed by section, only U1, representing 46.2 ± 1.4% of SPT, was strongly and significantly correlated with SPT. T and D times were not significantly correlated with SPT, although T1 was correlated with SPT when considered independently (Table 3).

Analysis of QF B yielded similar results: UT was strongly correlated with SPT (*r* = 0.79, *P* = 0.021), while T and D times were not. In QF A, no significant correlations were observed between U, T, or D times and SPT.

**Figure 3:**
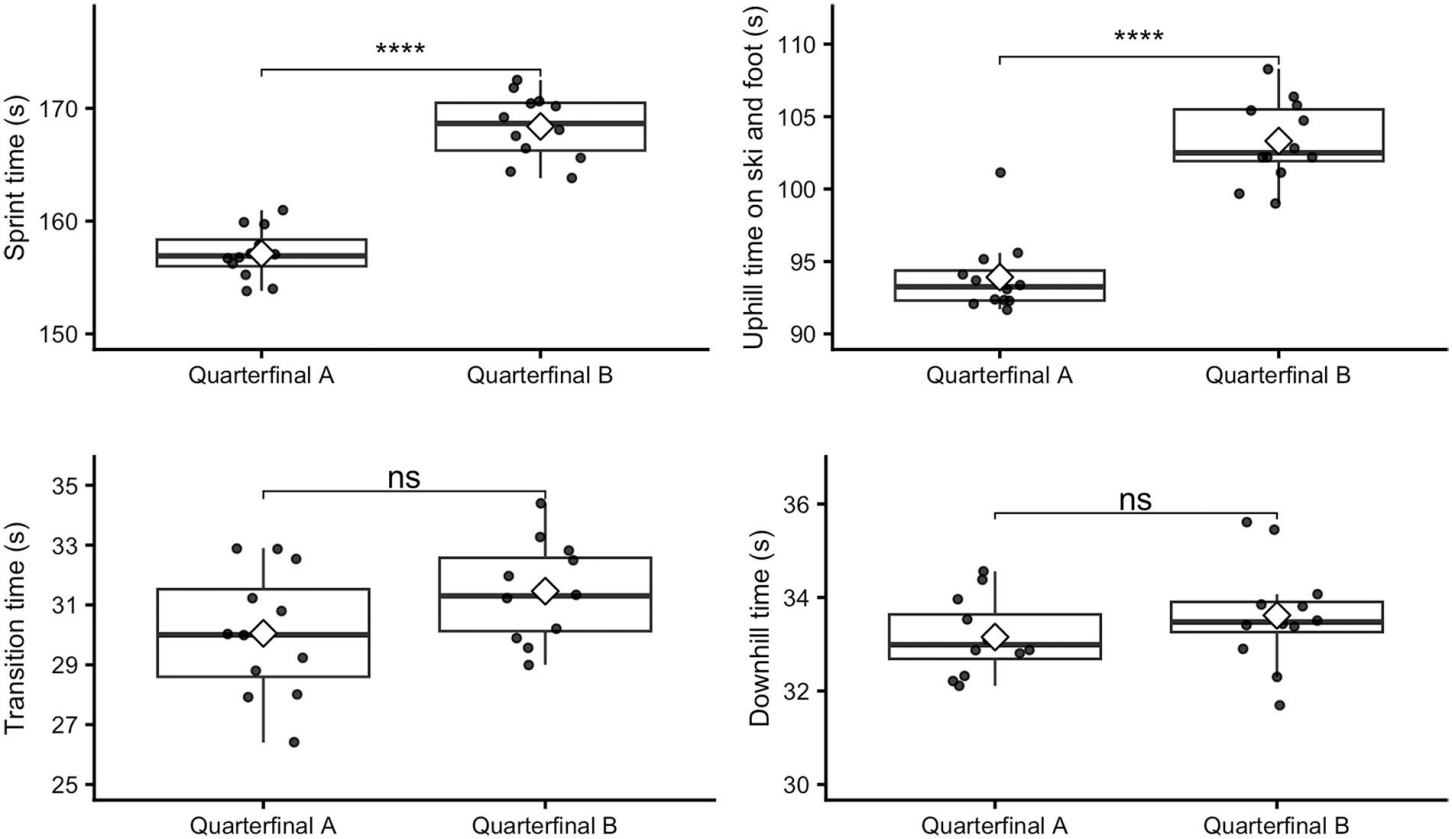
Box plot showing athlete variability by section and quarterfinal in 24 elite male ski mountaineering athletes who subsequently qualify for the semifinal. Total sprint time. Uphill time on ski and foot. Transition time. Downhill time. *P < 0.05, **P < 0.01, ***P < 0.001, ****P < 0.0001.

The interquartile range of UT was 92.3–94.4 s in QF A and 101.9–105.5 s in QF B. For T time, it was 28.6–31.5 s in QF A and 30.1–32.6 s in QF B (Figure 3).

The analysis of split-time contributions to SPT variability and the temporal contribution of each section, expressed as a percentage of SPT, is presented in Table 4 for QF A, QF B, and the entire sample. The contribution of T time to SPT variability appears to be higher in QFA than in QFB, differing by 28 percentage points.

**Table 4:** Relative contribution of race sections during the quarterfinals in 24 elite male ski mountaineering athletes who subsequently qualify for the semi-final.

Variable	Uphill section (U1 + U2 + U3)	Transition (T1 + T2 + T3)	Downhill (D)
Total (*n* = 24)			
Sprint time percentage (%)	60.1 ± 1.4	18.9 ± 1.1	20.5 ± 0.9
Relative contribution to sprint time variability (%)	81.5	15.2	3.4
Quarterfinal A (*n* = 12)			
Sprint time percentage (%)	59.8 ± 1.4	19.1 ± 1.2	21.1 ± 0.7
Relative contribution to sprint time variability (%)	62.2	46.8	−9
Quarterfinal B (*n* = 12)			
Sprint time percentage (%)	61.4 ± 1.1	18.7 ± 0.9	20.0 ± 0.7
Relative contribution to sprint time variability (%)	77	18.4	4.6

### 3.2 Laboratory-based determinants of uphill ski and foot section performance

**Figure 4:**
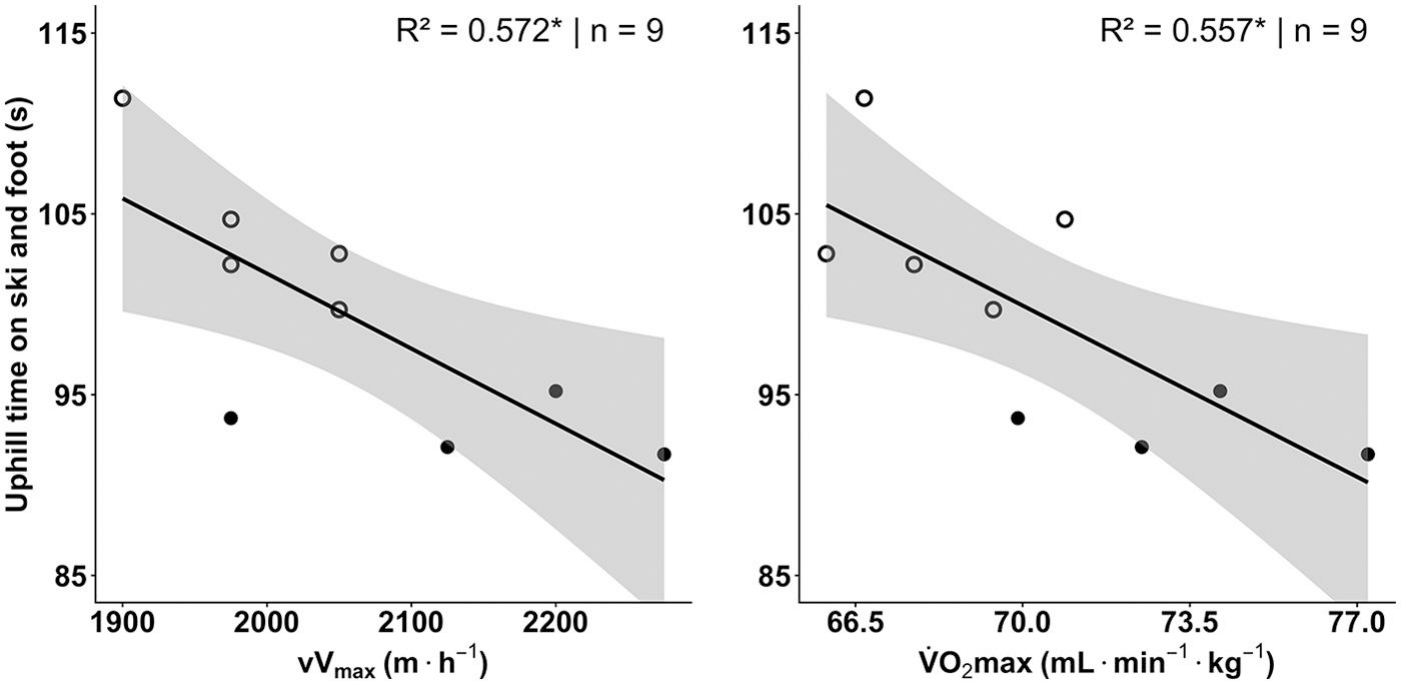
Quarterfinals uphill time on the ski and foot sections in relation to laboratory performance variables in nine world Cup ski mountaineering athletes: maximal vertical velocity (vV_max_) and maximal oxygen uptake (V̇O_2max_). Each data point represents an individual athlete, and lines were obtained using linear regression. Filled circles represent athletes selected for Draw A, whereas open circles represent athletes competing in Draw B.

For the laboratory-tested athletes (*n* = 9), mean final placement in the OSTE was 15 ± 11 (2–35), and mean UT was 99.3 ± 6.7 s (91.7–111.4). UT in QF was strongly and significantly correlated with vV_max_, (Figure 4) with the U ski segments (U1 and U3) driving this relationship, whereas the foot segment (U2) was not correlated.

Similarly, UT in QF was strongly and significantly correlated with $\dot{V}O_{2\max}$, (Figure 4) mainly due to U1 while the foot segment and the last ski section (U2 and U3) showed no significant correlation. Moderate associations were observed between UT in QF and both BLa_max_ and BLa_postTTE_, although these did not reach statistical significance (*r* = −0.54, *P* = 0.135; *r* = −0.50, *P* = 0.167, respectively) (Table 5). No significant correlation was observed between UT in QF and TTE.

**Table 5:** Correlation of physiological parameters with quarterfinals uphill time on ski and foot section (U1 + U2 + U3) and with each uphill section analyzed independently.

Parameters	N	Correlations *(r)*			
		UT	U1	U2	U3
$\dot{V}O_{2\max}$ *test*					
$\dot{V}O_{2\max}$ (mL.min^−1^.kg^−1^)	9	**−0**.746*	**−0**.728*	−0.533	−0.654
vV_max_ (m.h^−1^)	9	**−0**.757*	**−0**.681*	−0.629	**−0**.787*
BLa_max_ (mmol.L^−1^)	9	−0.539	−0.492	−0.548	−0.348
ΔBLa_rec_ (mmol.L^−1^)	9	−0.283	−0.367	−0.283	−0.184
ΔBLa_rec_ (%)	9	0.085	0.027	0.277	0.036
TTE test					
TTE test time (s)	9	−0.078	0.030	−0.270	−0.208
BLa_preTTE_ (mmol.L^−1^)	9	−0.335	−0.290	−0.419	−0.156
BLa_postTTE_ (mmol.L^−1^)	9	−0.504	−0.491	−0.425	−0.327
ΔBLa_TTE_ (mmol.L^−1^)	9	−0.330	−0.385	0.044	−0.322

UT, uphill time on ski and foot section; U1, first uphill skiing section; U2, foot section; U3, final uphill skiing section; vV, vertical velocity; V̇O_2_, oxygen uptake; BLa, blood lactate concentration; ΔBLa_rec_, 20 min passive lactate recovery; Max, maximal effort; TTE, time to exhaustion; BLa_preTTE_, blood lactate concentration before TTE test; BLa_postTTE_, blood lactate concentration after TTE test; ΔBLa_TTE_, blood lactate increase during the TTE test.

Bold values indicate statistical significance.

* *P* < 0.05.

Stepwise multiple regression analysis employing UT performance as the dependent variable revealed that $\dot{V}O_{2\max}$ and ΔBLa_rec_, in combination, provided the best prediction of this (UT; *R*^2^ = 0.82, *P* = 0.006)*.* The corresponding linear regression formulas, with non-standardized [and standardized] coefficients, were as follows:

$$\mathrm{UT}\;(s)=234.026-1.652\;[0.911]\times \dot{V}O_{2\max}\;(\mathrm{ml}.\mathrm{mi}n^{-1}.kg^{-1})-3.303\;[0.541]\;\times \;\Delta \mathrm{BL}a_{rec}\;(\mathrm{mmol}.L^{-1})$$

Both $\dot{V}O_{2\max}$ and ΔBLa_rec_ coefficient were statistically significant coefficients (*P* = 0.002 and *P* = 0.024, respectively) and were not affected by multicollinearity (VIF = 1.102). Furthermore the 95% confidence intervals (CI) did not cross zero for either $\dot{V}O_{2\max}$ (−2.45 to −0.85) or ΔBLa_rec_ (−6.00 to −0.60), indicating negative associations with UT.

According to a sensitivity analysis, the relative importance of the parameters in this model was 62.8% for $\dot{V}O_{2\max}$ and 37.3% for ΔBLa_rec_.

## 4 Discussion

The main findings of the present study were as follows:

1. U time was identified as the main determinant of SPT, as confirmed by the analysis of split-time contributions to SPT variability.
2. The first part of the sprint was found to be particularly influential in explaining SPT, highlighting the association of early-race performance and SPT.
3. T time contributed more to SPT variability in QF A than in QF B, suggesting its particular importance for the world-class ski mountaineers one year before the Olympic Games.
4. Lastly, $\dot{V}O_{2\max}$ and vV_max_ were the strongest laboratory predictors of UT. However, ΔBLa_rec_ also became a significant predictor when considered together with $\dot{V}O_{2\max}$ in a stepwise multiple regression model.

### 4.1 High importance of uphill time to explain ski mountaineering sprint performance

Most of total race time was spent in U sections (∼60%), with U1 alone accounting for nearly half of total SPT, whereas T and D sections represented ∼19% and ∼21%, respectively (Table 4). U time explained the largest share of SPT variability, while T time contributed a smaller but meaningful portion, and D time only marginally. Consistently, only U time differed significantly between QF A and QF B, whereas no between-group differences were observed for T or D times (Figure 3), highlighting the dominant role of U performance in qualifying for QF A among the 18 best athletes. These results agree with those reported by Fornasiero et al. (4), who showed that U sections account for most of sprint race time and performance variance; however, variance contributions in that study were estimated using stepwise multiple regression, whereas the present study used the covariance-to-variance ratio. Overall, these results, together with the limited sprint-specific competition data available in the literature (4, 6), provide converging evidence that U performance is a major determinant of sprint performance.

### 4.2 Major performance differences appear to be established early in the race

Total U time was strongly associated with SPT (Table 3). However, section-level analysis showed that this relationship was largely driven by U1, despite accounting for only ∼46% of total race time. T1, although a small fraction of the race, was also significantly correlated with SPT, whereas later U, D, and T sections showed no significant associations. These findings suggest that much of the performance differences are established during the first half of the race.

This pattern is consistent with a “fast-start” pacing strategy observed in supramaximal efforts such as the 800 m in track and field (18), 2,000 m rowing (19), 500–3,000 m cycling (20), and speed skating (21). Such a pacing profile has been proposed to enhance supramaximal performance by mobilizing the aerobic system more rapidly, thereby optimizing overall energy contribution (22). In SkiMo, however, early pacing is also likely influenced by tactical considerations: a fast start enables athletes to secure advantageous positions and select optimal racing lines. This aligns with Jones et al. (23), who showed in track and field that winners are often those who optimize positioning and avoid covering extra distance, rather than those with the highest average speed. The tactical aspect in SkiMo appears even more decisive, as positioning not only minimizes distance but also allows athletes to avoid technical or equipment constraints imposed by opponents and enter T1 in a favorable position to execute a fast T. Interestingly, in cross-country skiing sprints with a similar race format and equipment, even though there are no T, Andersson et al. (24) found that 61% of race winners were already leading after 30 m, which aligns with our findings. Thus, adopting a fast-start strategy in sprint SkiMo likely contributes to overall performance through both physiological and tactical mechanisms.

### 4.3 Transition time contributed more to sprint performance among the world's top ski mountaineering sprinters

As discussed above, U sections explain the majority of performance differences in sprint. Nevertheless, when examining the relative contribution of race sections to inter-individual variability in SPT, a more nuanced pattern emerged among the highest-performing athletes. While U time still accounted for the largest proportion of SPT variability in QF A, its contribution was lower than in QF B (62.2% vs. 77%). At the same time, T time explained a larger proportion of SPT variability in QF A than in QF B (46.8% vs. 18.4%), suggesting a partial shift in the contribution to SPT variability from U to T (Table 4). This observation is further supported by the larger interquartile range observed for T time compared with U time within QF A, despite T time representing only ∼20% of total race duration. This redistribution of section importance in explaining performance among the world's top sprinters suggests that climbing capacity tends to converge at this level of competition, making speed and execution quality during transitions a more discriminating factor for performance.

Previous studies reported moderate to large correlations between T duration and total sprint performance in SkiMo (*r* = 0.52–0.85) (5), and strong correlations in male athletes across competitions (*r* = 0.83–0.89) (4), indicating that T are generally relevant to sprint performance. However, these studies focused on overall associations without examining how the importance of T varies across performance levels. To our knowledge, this study is the first to compare T contribution to inter-individual variability between athletes ranked 1–18 and 19–36 in WC sprint qualifications, providing novel evidence that T performance becomes relatively more important among the very best SkiMo sprinters.

### 4.4 Aerobic capacity strongly predicts performance on sprint uphill ski and foot sections

As shown above, UT explained most of the variance in SPT; therefore, associations with physiological variables were examined using the UT in QF, which isolates U performance by excluding T times. Laboratory measures showed that both maximal aerobic velocity and body mass normalized $\dot{V}O_{2\max}$ were the strongest single predictors of UT (*R*^2^ = 0.572 and 0.557; *r* = −0.757 and −0.746, respectively). Moreover, stepwise multiple regression identified $\dot{V}O_{2\max}$ and ΔBLa_rec_ as the best combined predictors of UT, with $\dot{V}O_{2\max}$ alone accounting for 62.8% of the explained variance.

These findings are novel in elite SkiMo athletes, as previous studies only reported a moderate-to-strong correlation between absolute $\dot{V}O_{2\max}$ and uphill sprint skiing performance in men, with no such association observed in female athletes (6). In contrast, it seems consistent with previous work on cross-country skiing, reporting moderate-to-strong relationships between relative $\dot{V}O_{2\max}$ and sprint performance (7, 8, 25), as well as better maintenance of performance across repeated heats in athletes with higher aerobic capacity (9).

From a physiological perspective, this strong association was expected given the UT duration (∼90–120 s), for which aerobic metabolism provides the majority of the energy supply. Indeed, aerobic contribution has been estimated at ∼65% for efforts of ∼120 s (26), and ∼66% during 800 m running in elite middle-distance runners (mean duration: 113 s) (27). Together, these data support the interpretation that aerobic capacity is a primary determinant of performance in sprint U sections of SkiMo, despite the high-intensity and short-duration nature of these efforts.

In the present study, anaerobic capacity did not emerge as a significant predictor of UT, as neither BLa_max_ nor TTE showed significant correlations with UT. Nevertheless, previous literature indicates that anaerobic capacity does contribute to performance in cross-country skiing (9). However, consistent with other studies (6–8), we could not demonstrate a significant relationship between BLa_max_ and UT in our sample. This is likely due to the small sample size of elite athletes and the comparatively lower contribution of anaerobic capacity to ∼90–120 s maximal U efforts, in contrast to the dominant influence of $\dot{V}O_{2\max}$.

### 4.5 A higher passive blood lactate recovery seems to be associated with higher sprint performance on uphill ski and foot sections

In the stepwise multiple regression model including $\dot{V}O_{2\max}$ and ΔBLa_rec_, the latter, considered a practical proxy of lactate clearance capacity, explained a significant proportion of UT variance (37.3%). This result suggests that the capacity to clear lactate after a maximal exercise varies between athletes and contributes meaningfully to the ability to perform supramaximal efforts. However, given the relatively wide 95% CI for the ΔBLa_rec_ coefficient in the multiple regression model and considering the small sample size (*n* = 9) used in the regression analysis, these results should be interpreted with caution. Replication in a larger cohort is required.

Despite this limitation, this preliminary result is consistent with previous findings from Watanabe et al. and Messonnier et al. (28, 29), who showed that lactate exchange and removal capacities influence performance across different supramaximal efforts, including 800 m running and 2,500 m all-out rowing. Collectively, these findings suggest that lactate exchange and removal capacities are not merely indicators of recovery between exercises, but key determinants of supramaximal performance.

Based on previous studies, it can be hypothesized that the explanation of the relationship between lactate kinetics and supramaximal performance may be partly related to underlying muscular characteristics. Indeed, from a physiological perspective, lactate kinetics has been linked to multiple peripheral muscular traits. Passive lactate removal capacity, for instance, correlates with both the abundance of monocarboxylate transporters in skeletal muscle (30, 31) and their haplotypes (32). It is also associated with superior peripheral oxidative capacity (33) and with muscle capillarization density (34). While other tissues and mechanisms also contribute to lactate clearance, such as oxidative utilization by the heart and brain, or gluconeogenesis in the liver, skeletal muscle remains the predominant site of lactate removal (35). Therefore, despite the integrative and systemic nature of lactate kinetics, lactate removal capacity can reasonably be considered a proxy for muscular characteristics, encompassing notably monocarboxylate transporter characteristics, oxidative capacity, and perfusion.

Thus, it is physiologically plausible to suggest that passive lactate removal, which may serve as an indirect marker of peripheral muscle efficiency, can contribute to enhanced sprint performance during U skiing and foot sections in SkiMo.

### 4.6 Strengths and limitations

Data were collected during a pre-Olympic competition on the official Olympic course, ensuring high ecological validity, with a sample comprising elite athletes, including Olympic medalists and World Champions. However, the primary strength of this study lies in the unique race format of the OSTE competition, which enabled comparison between the top 18 and the bottom 18 competing athletes, and inclusion of more participants in the quarterfinals (36 vs. 30 in usual World-level competitions). Consequently, QF performance was used for analysis, providing a more representative measure than the time-trial approaches typically used in SkiMo World Cup studies, where athletes are not exposed to the same competitive pressure.

Several limitations should be noted. The first is that laboratory testing was conducted mid-June 2024, resulting in a time gap of approximately eight months between the laboratory assessments and the OSTE competition. This gap may have affected the observed associations, as physiological parameters can vary across the training season. Longitudinal observations in world-class rowers (36), showed seasonal variations in $\dot{V}O_{2\max}$ and power output, reflecting physiological adaptations across training phases and the competition calendar. However, these seasonal changes tend to evolve in a similar direction across athletes within the same training cycle. In the present study, all tested athletes followed a similar seasonal training structure, with a preparatory period during the summer and their main competitive goal in winter. This suggests that, although athletes were not in peak competitive form during laboratory testing, this lower-performance state is likely to have been comparable across participants and their individual evolution during this period were likely following the same direction. Nevertheless, small inter-individual variability cannot be ruled out.

The small sample size (*n* = 9) represents a further limitation, and therefore the multiple regression analyses should be viewed as exploratory and interpreted with caution. Although both 95% CI exclude zero, suggesting a significant negative effect, the ΔBLarec 95% CI (−6.00 to −0.60) is relatively wide, indicating greater uncertainty in the estimate despite its statistical significance. By essence elite athletes are exceptions, although a larger cohort would strengthen the statistical approach, it would also mean including athletes from different levels.

Finally, the analysis was based on a single competition, and results could differ in another event. Nevertheless, the strong association between SPT in the QF and ISMF sprint points (*r* = −0.77, *P* < 0.001) indicates that, overall, the fastest athletes in OSTE QF were also the top performers across the season.

## 5 Conclusion

In conclusion, (a) U performance is the main driver of sprint outcomes in elite ski mountaineering, (b) with the first skiing section and transition playing a critical role, highlighting the importance of a fast start. (c) Among the world's best athletes, speed and execution quality during transitions becomes a more decisive determinant of sprint performance, likely related to a homogenization of physical performance levels among these athletes. (d) Laboratory results highlighted the importance of aerobic power and lactate clearance in discriminating sprint performance. SkiMo sprinters therefore appear to require a combination of high aerobic capacity, effective lactate removal, explosive starts, and precise technical execution to compete with the world's best.

## Data Availability

The raw data supporting the conclusions of this article will be made available by the authors, without undue reservation.
